# Docosahexaenoic Acid (DHA) Deficiency in Breast Milk: A Cross-Sectional Study of Lactating Women in Serbia, Montenegro, and North Macedonia

**DOI:** 10.3390/nu18172761

**Published:** 2026-08-24

**Authors:** Tena Niseteo, Ana Ilić, Nevena Cupara, Dijana Đurović, Maja Dimitrovska, Lidija Poček, Marina Pop Lazarova, Jelena Martić

**Affiliations:** 1 Referral Center for Pediatric Gastroenterology and Nutrition, Children’s Hospital Zagreb, 10000 Zagreb, Croatia; 2 Department of Nutrition, Food Quality and Safety, Faculty of Food Technology and Biotechnology, University of Zagreb, 10000 Zagreb, Croatia; ana.ilic@pbf.unizg.hr; 3 Faculty od Technology, University of Montenegro, 2 Cetinjski Put, 81000 Podgorica, Montenegro; 4 Center for Ecotoxicological Research, Sarla de Gola 2, 81000 Podgorica, Montenegro; 5 Department of Food Quality and Safety, Institute for Public Health of the Republic of N. Macedonia, 50th Division No. 6, 1000 Skopje, North Macedonia; 6 Institute for Children’s Diseases, Clinical Center of Montenegro, 81000 Podgorica, Montenegro; 7 PHI General Hospital “Re-Medika”, 1000 Skopje, North Macedonia; 8 Medical Faculty, University of Belgrade, 11000 Belgrade, Serbia; 9 Mother and Child Health Care Institute of Serbia “Dr Vukan Cupic”, 11000 Belgrade, Serbia

**Keywords:** DHA, *n*-3 PUFA, lactation, breast milk, infants, dietary intake

## Abstract

**Background/Objectives**: Docosahexaenoic acid (DHA) is an essential component of breast milk for infant neurocognitive and visual development. Because DHA depends largely on dietary intake, women with low seafood consumption are at increased risk of inadequate DHA contents in breast milk. This study aims to quantify the DHA content in breast milk and assess how recommended dietary supplementation during lactation affects these contents among lactating women in three Balkan countries. **Methods**: This cross-sectional multicenter study included 128 lactating women (18–35 years). Dietary intake of DHA was assessed using a modified Food Frequency Questionnaire. DHA in breast milk was measured by gas chromatography following standard protocols. Both the FFQ and breast milk samples were collected at two time points: 20–40 (visit 1—V1) and 80–100 days (visit2—V2) postpartum. **Results**: In lactating women, seafood intake was low (Visit 1: 37.5 (0.0–93.8) g/week), meeting about 28% of the recommended amount, with some variation between countries. Consequently, the mean dietary *n*-3 PUFA and DHA intake for the total sample was less than 50% and 5% of the recommended daily allowance, respectively. Correlation analysis indicates that DHA content in breast milk is related to dietary DHA intake (Visit 1: r = 0.258, *p* = 0.001; Visit 2: r = 0.238, *p* = 0.012). DHA contents failed to reach the 0.3% clinical threshold for optimal neurodevelopment, with average levels ranging from 0.149 to 0.165%. Furthermore, no significant differences were observed between supplemented and unsupplemented women at either time point, highlighting a persistent regional deficit regardless of supplementation status. **Conclusions**: Lactating women in the Balkan region have a low dietary intake of *n*-3 PUFA and DHA, which is reflected in the breast milk lipid profile, regardless of standard supplementation. These findings highlight the need for a targeted nutritional strategy both via nutrition and supplementation to improve DHA intake in lactating women, but more importantly in infants.

## 1. Introduction

Human breast milk is a complex biological fluid whose composition is dynamically influenced by maternal nutritional status, time postpartum, and circadian rhythms. It typically contains 87–88% water and 124 g/L of solids, providing an average energy density of 65–70 kcal/100 mL. Lipids are the primary energy source, accounting for approximately 50% of this density, followed by carbohydrates (40%) and proteins [1]. The protein fraction undergoes a distinct transition from a 90:10 whey-to-casein ratio in colostrum to 60:40 in mature milk [2].

Lipids in breast milk are critical for infant energy intake and central nervous system development [3]. The relationship between maternal DHA status and infant neurodevelopment is well established. DHA constitutes approximately 15–20% of total fatty acids in the cerebral cortex and 30–40% of those in the retina, where it is incorporated during the third trimester and first two years of life, when it is the period of peak synaptic proliferation and myelination [4]. Epidemiological studies consistently associate low maternal DHA intake and low breast milk DHA content with poorer visual acuity, reduced cognitive scores, and lower language development indices in infancy [5,6].

While the content of palmitic and oleic acids is relatively stable, docosahexaenoic acid (DHA) levels are highly sensitive to maternal diet. Research indicates that a minimum threshold of 0.3% DHA of total fatty acids is required for optimal cognitive and visual development [7]. Evidence suggests that the intake of eicosapentaenoic acid (EPA) and DHA has potential benefits such as an improvement in fetal neurodevelopment and reduced risks of pre-eclampsia and metabolic diseases, in addition to effects on gestational duration and birth weight.

However, significant global variations exist; countries with high marine intake, such as Japan, maintain DHA levels in breast milk up to 1.4%, whereas levels in Central and Eastern Europe often drop to 0.15–0.25% [8].

In contrast, arachidonic acid (ARA) levels remain relatively stable due to metabolic regulation and mobilization from maternal adipose stores. While DHA levels directly reflect acute dietary intake or supplementation (200–600 mg/day), ARA levels are less influenced by external dietary fluctuations [9].

The secretion of DHA into breast milk involves the uptake of dietary lipids via chylomicrons and the subsequent transport of free fatty acids bound to albumin in the plasma. Within the mammary epithelium, DHA is activated into DHA-CoA by acyl-CoA synthetase and esterified into milk fat globules. This process is facilitated by fatty acid-binding proteins, which maintain the availability of long-chain polyunsaturated fatty acids for triglyceride synthesis [10].

Research highlights significant variability in breast milk DHA content globally, driven by diet, geography, and socioeconomic status [11]. While fish-rich diets correlate with higher DHA levels, many low-to-middle-income countries fall below the thresholds necessary for optimal neurocognitive development [12].

Despite global trends, data remains scarce for non-EU countries in Southeast Europe, particularly regarding the nutritional status of breastfeeding mothers—a highly sensitive demographic. Given the unique regional dietary patterns and internal variations, systematic research is essential to assess DHA levels in breast milk and identify potential nutritional risks in this population [13].

Dietary supplementation during the prenatal and lactating periods is frequently recommended to childbearing and lactating women. Many of these formulations contain DHA, typically at a standardized dose of 200 mg per day. While several studies have demonstrated a positive effect on maternal milk DHA levels, evidence suggests that this impact is highly dose-dependent [7,14].

The main objective of this study is to quantify the DHA content in breast milk of lactating women from three distinct Balkan populations across key postpartum stages (at the first and third months of life), and to cross-reference these lipid levels with maternal dietary patterns captured via a Food Frequency Questionnaire (FFQ). By evaluating the relationship between habitual diets, supplementation practices, and actual breast milk composition, this study aims to clarify whether current regional dietary habits support the recommended 0.3% DHA threshold in mothers’ milk, thereby providing a baseline to discuss the potential need for nutritional recommendations.

## 2. Materials and Methods

### 2.1. Study Design and Participant Recruitment

This cross-sectional multicenter study was conducted between February 2024 and February 2025 to evaluate the lipid profile of mature breast milk in the Balkan region. A total of 128 lactating women were recruited from Serbia (*n* = 36), Montenegro (*n* = 55), and North Macedonia (*n* = 37). Inclusion criteria required participants to be aged 18–35 years, have delivered a healthy full-term infant (37–41 weeks’ gestation), and to exclusively breastfeed. Exclusion criteria included maternal chronic disease and use of chronic medication. All participants provided written informed consent prior to enrolment. The study was conducted in accordance with the Declaration of Helsinki and approved by the Ethics Committee in all three countries (Health Center “Dr Simo Milošević”, Accredited Healthcare Institution—No: 4935/7, University of Montenegro, Faculty of medicine, Committee for medical ethics and bioethics—No: 126/3; Cyril and Methodius University in Skopje, Faculty of Medicine—No: 03-5505/3).

Financial support for this study was provided by 4U Pharma GmbH, Herisau, Switzerland, which covered the costs of sample transport and laboratory analysis. The company had no role in the study design, data collection, statistical analysis, or the interpretation of the results.

### 2.2. Data Collection Protocol

**Figure 1:**
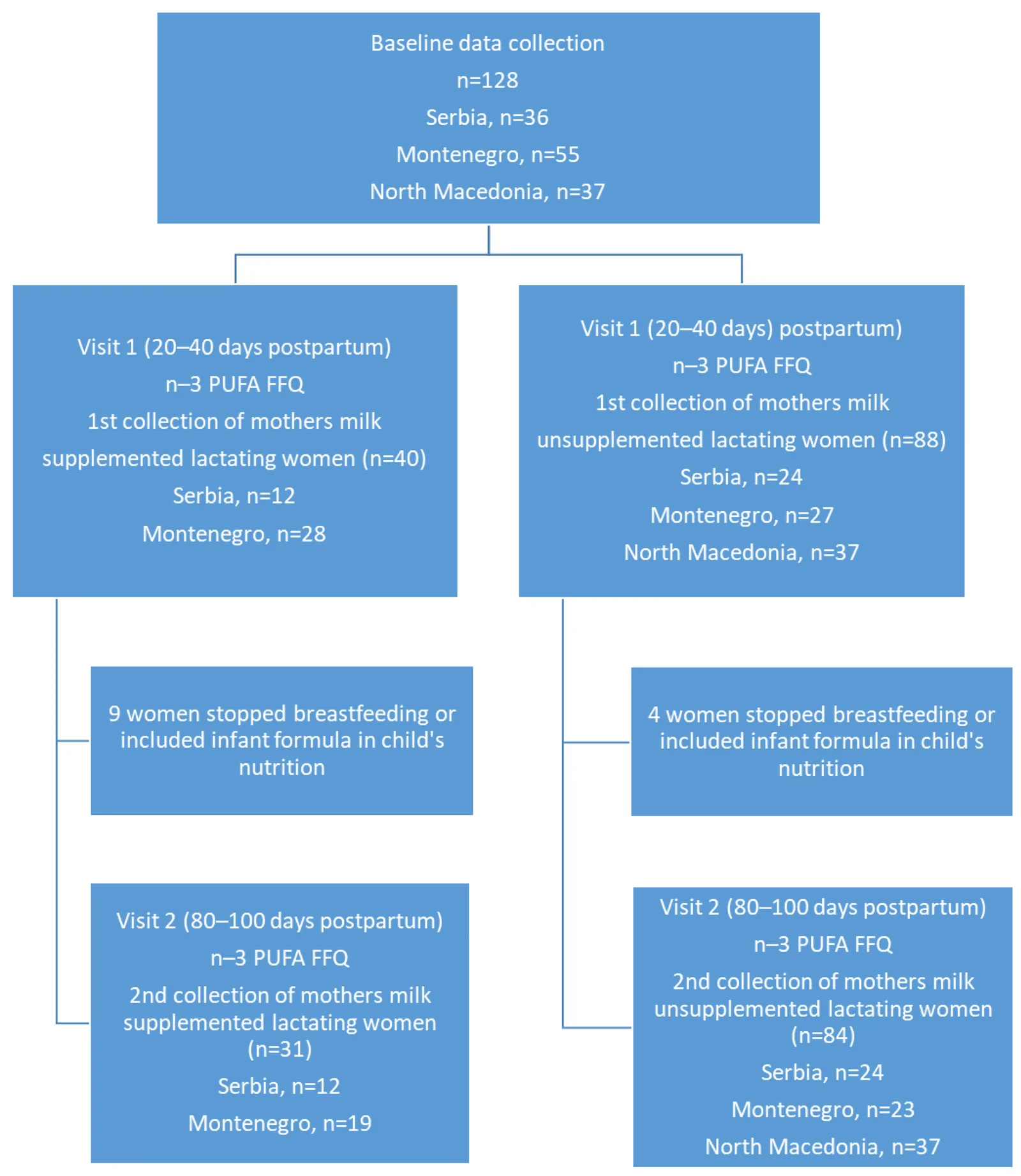
Study flow diagram.

Data collection was facilitated by community nurses across three scheduled visits to ensure longitudinal consistency: the initial visit (Visit 0, V0) was within less than 10 days postpartum. During this visit, baseline demographic and medical history data were recorded, including maternal and neonatal anthropometric measurements using standard protocols (body weight; body height for women; and body weight and length for neonates). The body mass index (BMI) of women was calculated from the measurements obtained and classified according to WHO classification [15]. First visit (Visit 1, V1) for collection of breast milk was conducted between the 20th and 40th day postpartum. During this visit, administration of a modified Food Frequency Questionnaire (FFQ) and collection of the first mature milk sample was done. The second visit (Visit 2, V2) was conducted between the 80th and 100th day postpartum. During the visit, data was collected by FFQ, and a second mature breast milk sample was collected (Figure 1).

### 2.3. Dietary Assessment

Nutritional habits were assessed using a modified FFQ specifically validated for omega-3 polyunsaturated fatty acid (*n*-3 PUFA) intake [16]. The tool was adapted to include regional fish species, seafood products, and plant-based omega-3 sources (walnuts, chia seeds, flaxseed oil) common in the Balkan diet. Intake frequency was quantified using a 10-point scale (ranging from “never” to “2 times daily”). Daily DHA intake (mg/day) was calculated by integrating frequency factors with standardized portion sizes and regional food composition databases [17,18]. Intakes of *n*-3 PUFA and DHA were compared with Dietary Reference Values from the European Food Safety Authority (EFSA) [19].

### 2.4. Milk Sampling and Lipid Analysis

To account for diurnal fluctuations in lipid content, a standardized sampling protocol was followed. Participants provided 40 mL of milk (20 mL collected between 06:00 and 12:00 h and 20 mL between 18:00 and 24:00 h). Milk was expressed by hand or breast pump after breastfeeding. Samples were immediately stored at −20 °C and, the day after, transported in temperature-controlled coolers to the laboratory and stored in deep-freeze units at −80 °C before analysis. Lipid analysis was performed using gas chromatography with flame ionization detection (GC-FID). Before analysis, samples underwent homogenization and alkaline transesterification with methanolic potassium hydroxide (2 mol/L) to produce fatty acid methyl esters (FAME) in accordance with ISO standards (12966-1, -2, -4) [20,21,22]. Chromatographic separation was achieved using a Shimadzu Nexis GC-2030 (Shimadzu Europa GmbH, Duisburg, Germany) equipped with an Rtx-2330 capillary column (105 m × 0.25 mm × 0.2 μm). The temperature program utilized a gradient from 80 °C to 240 °C with a 50:1 split ratio. Fatty acids were identified by comparison of retention times against the following certified reference standard mixtures. The primary identification standard used at all three laboratories was the Supelco 37 Component FAME Mix, Trace CERT^®^ (CRM47885, Sigma-Aldrich/Supelco, St. Louis, MO, USA), a certified reference mixture of 37 fatty acid methyl esters spanning C4:0 (butyric acid) to C22:6n-3 (DHA), and covering the full C4–C24 analytical range including saturated, monounsaturated, trans, and polyunsaturated fatty acids. At the North Macedonia laboratory, three supplementary standards were additionally used: C4–C24 Even Carbon Saturated FAMEs (Cat. No. 49453-U, Sigma-Aldrich) for additional confirmation of short-chain and medium-chain saturated fatty acids including C4:0 and C6:0; Linoleic Acid (C18:2) Methyl Ester Mix cis/trans (CRM47791, Supelco) for identification of cis and trans C18:2 isomers; and Linolenic Acid (C18:3) Methyl Ester Mix cis/trans (CRM47792, Supelco) for identification of cis and trans C18:3 isomers. At the Montenegro laboratory, the equivalent Restek Food Industry FAME Mix 37 components (Cat. No. 35077, Restek, Bellefonte, PA, USA) were used as the primary standard, supplemented by a Restek Trans Fat Reference Standard (Cat. No. 35629) for identification of individual trans isomers including elaidic acid (C18:1 trans-9) and transvaccenic acid (C18:1 trans-11). The Serbian laboratory applied FAME standards covering the equivalent C4–C24 range per SRPS EN ISO 12966; fatty acids not appearing in Serbian results were below the limit of quantification in those samples. Results are expressed as a relative weight percentage (wt%) of the total identified fatty acids, calculated as x_i_ = (A_i_/ΣA) × 100, where A_i_ is the peak area of the individual fatty acid methyl ester, and ΣA is the sum of all identified FAME peak areas. Total *n*-3 PUFA was defined as the sum of ALA (C18:3n-3), eicosatrienoic acid (C20:3n-3), EPA (C20:5n-3) and DHA (C22:6n-3). A complete fatty acid profile for all participants is provided in Supplementary Table S1.

### 2.5. Statistical Analysis

Statistical analysis was conducted using SPSS software (IBM SPSS Statistics for Windows, version 23.0, Armonk, NY, USA: IBM Corp.). Descriptive data are presented as means and standard deviation or median (interquartile range), depending on distribution; categorical data are presented as frequencies or percentages. Due to the non-normal distribution of the nutritional data and the specific regional subgroup sizes, the Wilcoxon test was used to evaluate differences in dietary intake between visits, while the Mann–Whitney U test was used to assess differences in DHA content in breast milk between unsupplemented and supplemented lactating women. Spearman’s rank correlation was applied to examine the relationship between dietary DHA intake and breast milk DHA content. Statistical significance was defined as *p* < 0.05.

## 3. Results

The study included a total of 128 lactating women from Montenegro (42.9%), Serbia (28.1%), and North Macedonia (28.9%). On average, women were slightly overweight according to BMI (Table 1). In the total sample, most women rated their diet as good (57.5%) or very good (40.2%). Regarding DHA supplementation, 31.3% of women used it during pregnancy and lactation. The mean birth weight of neonates was 3472.7 ± 440.6 g, and the mean length was 52.0 ± 1.3 cm (Table 1).

### 3.1. Dietary Intake of Total n-3 PUFA and DHA

The study cohort (*n* = 128) demonstrated a chronically low intake of fish and seafood. At V1 (20–40 days postpartum), the mean intake in all was 37.5 (0.0–93.8) g/wk, representing only 17.4% (0.0–43.6%) of the 215 g/wk benchmark derived from EFSA guidelines [23]. By V2 (80–100 days postpartum), a non-significant increase was observed, indicating longitudinal stability in these low consumption patterns. Regional analysis identified North Macedonia as having the highest consumption (median 93.8; IQR 37.5–131.3 g/week at V2), whereas dietary intake of fish and seafood in lactating women in Montenegro and Serbia remained significantly lower, averaging approximately 37.5 (0.0–93.8) g/wk in both countries. Animal-derived sources (fish and seafood) were the primary contributors to omega-3 status, accounting for approximately 80% of total intake across the three nations. Plant-based sources (alpha-linolenic acid, ALA) contributed the remaining 20%. In Serbia, the reliance on animal sources was most pronounced (86%), while North Macedonia demonstrated a marginal shift toward plant-based sources by V2 (30% of total intake).

Consequently, the mean dietary *n*-3 PUFA intake for the total sample (median 91.3; IQR 17.2–227.5 mg/day) was less than 50% of the daily intake recommended by EFSA for lactating women (Table 2), with no differences between visits. Additionally, women had a mean intake of 3.0 (0.0–7.8) mg/day (V1) and 3.7 (0.0–11.1) mg/day (V2) of DHA, which is less than 5% of the DRV (200 mg per day). Country-specific analysis showed differences in *n*-3 PUFA and DHA intake from food, with women in North Macedonia having the highest intake, followed by women in Serbia and Montenegro. Significant differences were observed only in DHA intake among North Macedonian women, where it was significantly higher (*p* < 0.001) in V2 (median 9.7; IQR 2.9–18.7 mg/day) compared to V1 (median 4.8; IQR 1.8–8.8 mg/day).

### 3.2. DHA in Human Milk

The biochemical analysis of mature breast milk revealed critically low content of DHA. At V1, the median DHA content was 0.151% of total fatty acids, which further regressed to 0.130% by V2. Total *n*-3 PUFA content mirrored this downward trend, declining from 1.278% to 1.195%, respectively.

**Figure 2:**
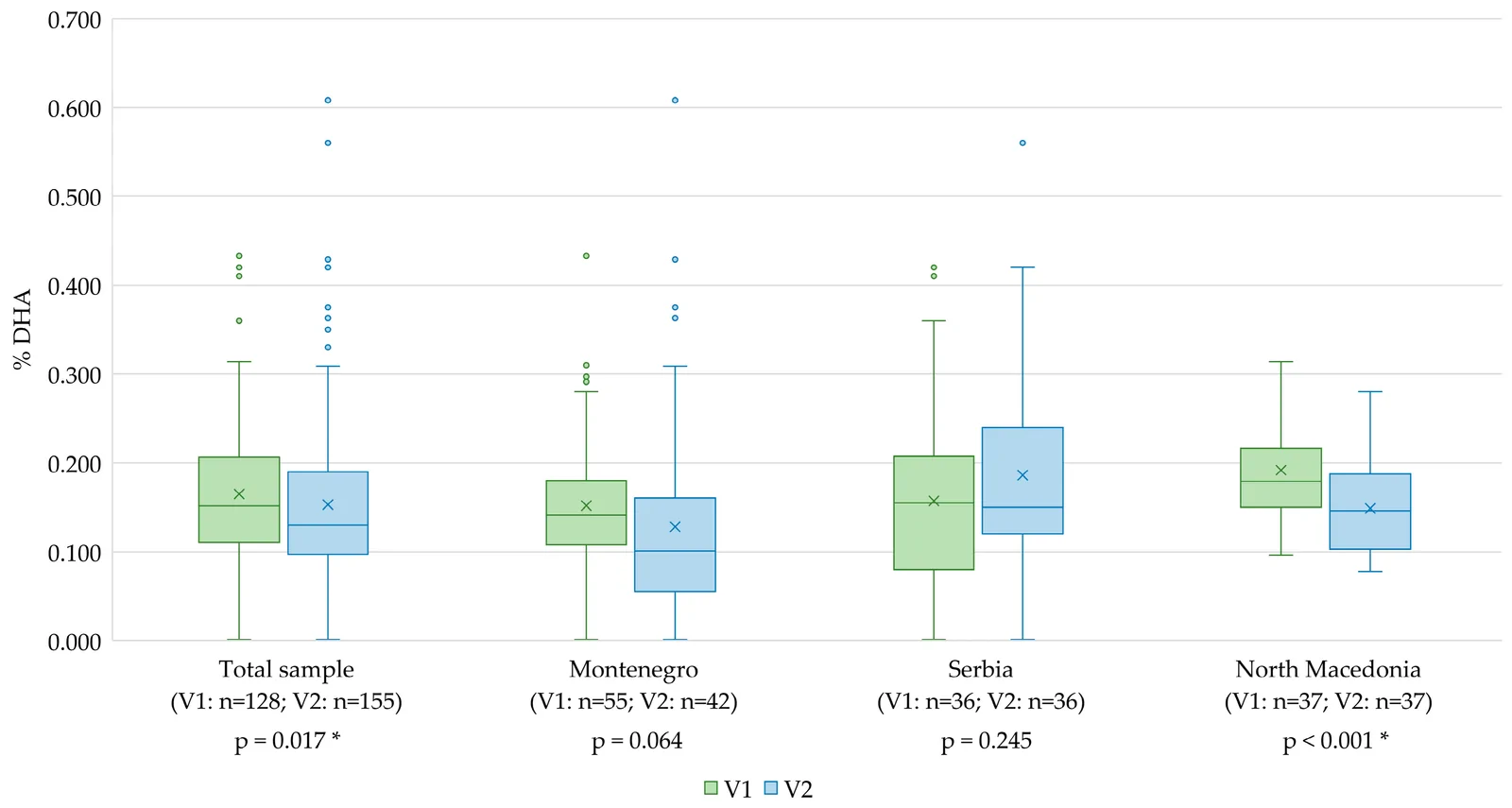
DHA content in breast milk. * Differences between visits were tested using the Wilcoxon test (*p* < 0.05).

Regional DHA content in breast milk closely correlated with dietary patterns. In lactating women who were not using DHA supplements, the content of DHA was 0.151% (0.112–0.202%) at the V1 time point, and at time point V2, it was 0.147% (0.111–0.262%). In North Macedonia, DHA content was highest reaching at V1 time point 0.181% (0.150–0.219%) declining in V2 to 0.147% (0.104–0.190%); in Montenegro, it was 0.138% (0.108–0.180%) at V1, declining to 0.101% (0.058–0.160%) at V2; and these values were lowest at time point V1 Serbia, 0.155 (0.080–0.205%), and 0.150% (0.120–0.024%) at time point V2 (Figure 2). No regional average reached the minimum clinical threshold of 0.3% DHA in mothers’ milk.

**Figure 3:**
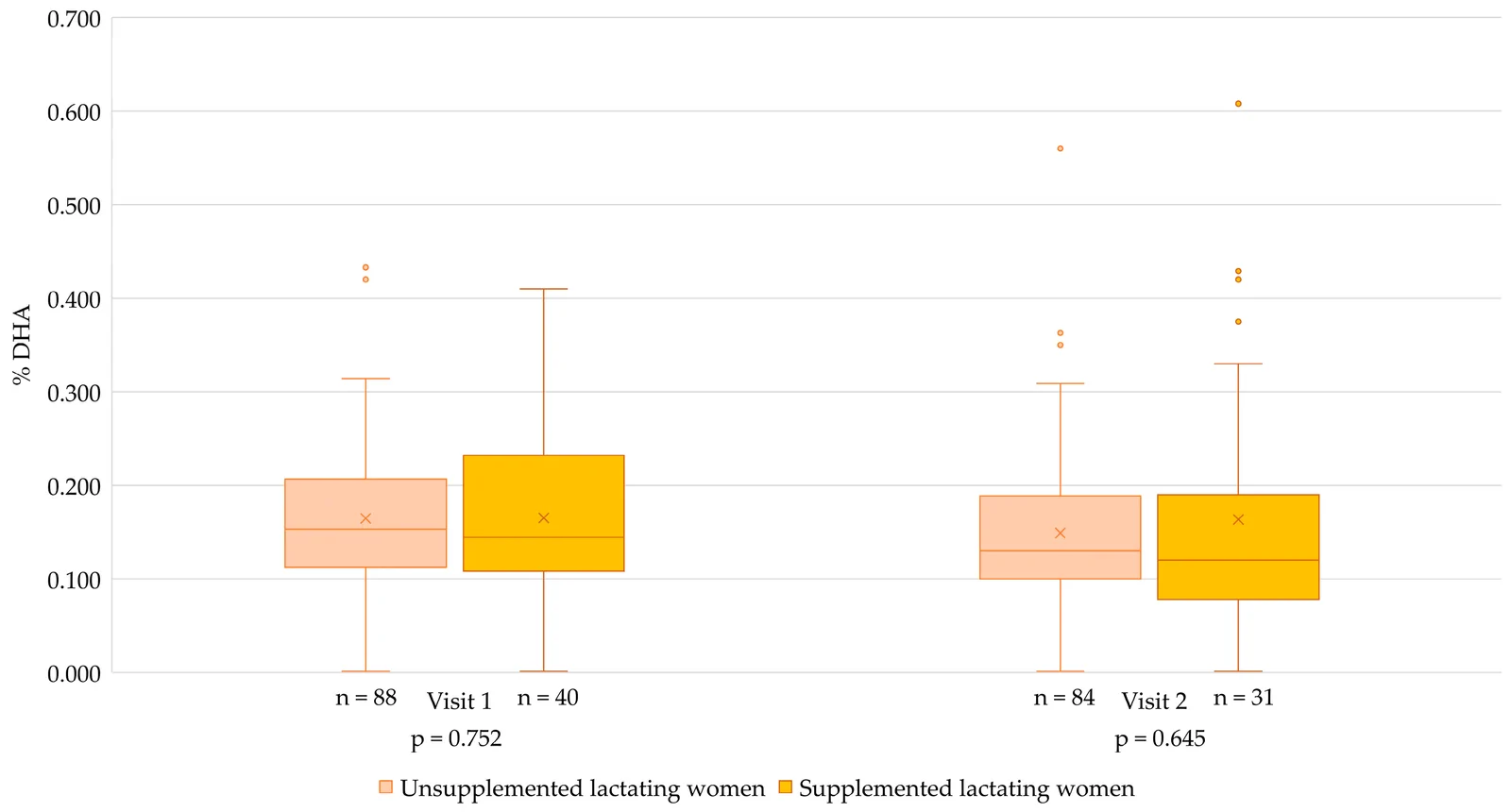
Differences in DHA content in breast milk of unsupplemented and supplemented lactating women. Differences were tested using the Mann–Whitney U test (*p* < 0.05).

There was no significant difference in DHA content in breast milk of supplemented (*n* = 40) and unsupplemented (*n* = 88) women at V1 and at V2 (supplemented, *n* = 31 and unsupplemented, *n* = 84). In unsupplemented women’s breast milk at V1, DHA content was 0.151% (0.112–0.202%), and in supplemented women’s breast milk, 0.147% (0.111–0.262%) (*p* = 0.752). At time point V2, DHA content was 0.130% (0.100–0.190%) in unsupplemented and 0.120% (0.081–0.190%) in supplemented women’s breast milk (*p* = 0.645) (Figure 3).

Linear correlations between dietary intake of DHA and DHA content in breast milk at two time points in the total sample, as well as stratified by country and supplementation status, are presented in Table 3. In the total sample, a weak but significant positive correlation was observed between dietary DHA intake and breast milk contents at both visits (V1: r = 0.258, *p* = 0.001; V2: r = 0.238, *p* = 0.012). When stratified by supplementation status, results in unsupplemented women showed a relationship between dietary intake and breast milk content at both visits (V1: r = 0.338, *p* = 0.002; V2: r = 0.277, *p* = 0.012), while in supplemented women, results showed this relationship only at V2 (r = 0.588, *p* = 0.044). Country-specific analysis revealed a relationship between high dietary DHA intake and breast milk content in unsupplemented women only in Montenegro at both visits (V1: r = 0.445, *p* = 0.020; V2: r = 0.414, *p* = 0.050), while in other countries, no correlation between these variables was observed.

## 4. Discussion

Our findings demonstrate a profound nutritional gap among lactating women in the Balkan region. The observed DHA levels are among the lowest reported in the European literature and fall significantly below the global median of 0.37% [11]. The low fish and seafood intake observed in Serbia (37.5 g/wk) aligns with findings by Milesevic et al. (2025) [24], who noted a general absence of seafood in the Serbian diet. However, the data from Montenegro presents a “tourism paradox,” while national EFSA databases suggest that 42% of pregnant women in Montenegro consumed fish with a mean intake of 96.42 g per day [25]; our specific cohort of lactating women consumed less than 50 g/wk. This discrepancy within the Montenegro population can be explained by the fact that national statistics are likely inflated by the tourism sector and do not accurately reflect the habitual dietary patterns of the local population. As low intake of fish is observed in all participants, consequently there is also a low dietary intake of omega-3 fatty acids.

The DHA contents identified in this study are strikingly similar to those reported in other middle-income nations with limited seafood access. A Hungarian study by Mihályi et al. (2015) included 87 healthy women with a mean age of 32.9 years [26]. At six weeks postpartum, 61 participants were breastfeeding; analysis of their breast milk composition revealed a DHA content of 0.14% of total fatty acids at 6th week postpartum. This exceptionally low DHA level suggests a limited intake of *n*-3 PUFA in the maternal diet during the early postnatal period. Similar results were found by a Brazilian study where, in 80 participants, DHA content in mature milk was measured twice (at the 4th week and 13th week of breastfeeding). The content of DHA was reported to be 0.14 ± 0.05% [27]. Compared to reports of the neighboring country Croatia (0.21%), the contents in our cohort are somewhat lower. Croatian study reported dietary seafood intake and breastmilk DHA content from a sample of women living on the Croatian seacoast to be on the lower side compared to other Mediterranean countries [13]. Substantially higher levels at 0.75–1% were observed in high-income Mediterranean countries like Italy [28,29]; however, the review by Fu et al. (2016) showed substantial differences within European countries, with the lowest DHA content in Hungary, Germany, and the Netherlands and the highest in Italy, Sweden, and Spain [12]. The review by Cervantes-De Celis et al. (2025) showed significant global variation in DHA levels, ranging from 0.17% to 0.99% [30]. Populations with the highest fish consumption, such as Japan, have approximately 1.1% DHA in breast milk, whereas the USA reported the lowest, at 0.17%.

More recent studies suggest that 0.3% of DHA content is the result of seafood (fish) consumption worldwide; however, in countries where seafood consumption is higher, like Japan, these contents are three times higher. This is why 0.3% of DHA is the minimum recommended DHA content for normal neurological development; moreover, the authors suggest that the optimal range for DHA content in breast milk lies between 0.5% and 1% [31].

The impact of maternal supplementation on the lipid profile of breast milk has not been extensively investigated. In this study, the supplemented group received a standard multivitamin product containing a standardized dose of 200 mg DHA. Our analysis revealed that this level of supplementation did not result in a statistically significant difference in milk DHA content when compared to unsupplemented lactating women. At the first visit (V1), the unsupplemented group exhibited a median content of 0.151% and the supplemented group of 0.147% (*p* = 0.752). By the second visit (V2), levels were 0.120% in the supplemented group and 0.130% in the unsupplemented group (*p* = 0.645), indicating that the standard 200 mg dose was insufficient to elevate milk levels toward the recommended 0.3% clinical threshold. These findings align with existing evidence, suggesting that the impact of supplementation is highly dose-dependent and often influenced by long-term nutritional status rather than acute intervention. Some research indicates that maternal milk DHA directly reflects dietary intake or supplementation, typically within a range of 200–400 mg/day [32]. However, emerging studies have questioned the efficacy of high-dose supplementation in increasing milk content. For instance, research conducted by Paquet et al. (2024) observed a significant increase in breastmilk DHA content after supplementation of mothers with 1.2 g of DHA per day [33]. Furthermore, several clinical observations have demonstrated that low-dose interventions may fail to produce significant changes. A study by Sherry et al. (2015) highlighted that while high-dose supplementation (800 mg) significantly raised levels, a lower dose of 200 mg provided more modest results, which in some populations may not reach statistical significance depending on the baseline deficiency [32].

Conversely, our results support the perspective that low-dose supplementation (200 mg) has a limited impact in populations with a “Westernized” dietary pattern and chronic omega-3 deficits. In our cohort, mean dietary omega-3 intake reached less than 50% of the recommended daily allowance, and fish consumption was only 17.4% of the EFSA benchmark [34].

The optimal range for breast milk DHA is considered to be between 0.5% and 1.0% [30]. Given that our participants failed to achieve even the “minimal” 0.3% threshold, there is a tangible risk to the neurocognitive and visual maturation of infants in these populations. The relative stability of ARA in breast milk, contrasted with the extreme dietary sensitivity of DHA, emphasizes that the latter cannot be adequately maintained through endogenous synthesis or adipose tissue mobilization alone.

Producing breast milk with the minimum 0.3% DHA required to meet an infant’s metabolic needs (100 mg/day), a maternal intake of at least 200 mg DHA is recommended. However, the benefits of supplementation versus enriched formula remain debated, as outcomes depend on DHA/ARA ratios, baseline status, and genetics [35]. Notably, infants fed enriched formulas may even exhibit lower rates of early-life infections and allergies than breastfed infants in populations where maternal DHA levels are deficient [36].

As earlier mentioned, the recommended daily intake of DHA for infants is 100 mg per day [19], whereas ESPGHAN (European Society for Paediatric Gastroenterology, Hepatology and Nutrition) recommends 35–65 mg/kg body weight/day [37]. The nutritional landscape regarding DHA in infant development has shifted significantly with the implementation of EU Regulation 2016/127 (effective as of 2020), which mandates that all infant formulas must contain 20–50 mg of DHA per 100 kcal. This regulatory change effectively sets a target level of 0.5% to 1% of total fatty acids as the optimal standard for neurodevelopmental support [38]. Therefore, in countries with low seafood consumption and an established low intake of *n*-3 PUFA, especially DHA among lactating women and low content of DHA in their mature milk, it would be wise to consider infant supplementation at the recommended dose of DHA (100 mg DHA per day).

The primary strength of this research lies in its multicenter, longitudinal design, which provides some of the first comparative data for Balkan countries. By tracking the milk lipid profile at two distinct stages of mature lactation and utilizing a standardized 24 h collection method—pooling samples from both day and night—we were able to account for the natural daily fluctuations that often make lipid levels difficult to measure accurately. Furthermore, by using a Food Frequency Questionnaire specifically tailored to regional food sources, it was possible to draw a clear, culturally relevant connection between local dietary habits and the resulting composition of the milk.

However, certain limitations must be noted. As an observational study, supplementation was self-reported and not randomized, and the lack of a supplemented cohort in North Macedonia restricted a full three-country comparison of supplementation efficacy. While the sample size was sufficient to identify significant regional trends, a larger, more diverse cohort would strengthen these findings. Additionally, as a limitation, the study did not assess maternal erythrocyte DHA levels or genetic polymorphisms (such as the FADS gene cluster), which are known to influence endogenous DHA synthesis and secretion. Furthermore, due to practical and financial constraints in a multicenter study, we did not include analysis of PUFA concentrations in maternal erythrocyte membranes. This analysis would have provided an objective biomarker of long-term *n*-3 PUFA status and allowed cross-validation of the FFQ-derived dietary estimates. Despite these limitations, the consistent evidence of a regional DHA deficit provides a robust foundation for future public health strategies and targeted nutritional interventions.

## 5. Conclusions

The chronic underconsumption of seafood among lactating women in Serbia, Montenegro, and North Macedonia directly translates to a suboptimal lipid profile in breast milk. Standard supplementation of breastfeeding women did not achieve a significant rise in DHA in breast milk. To mitigate potential developmental delays in breastfed infants, regional public health strategies must prioritize omega-3 education and consider the implementation of routine DHA supplementation protocols for infants during the first 1000 days of life.

## Figures and Tables

**Table 1:** Demographic and anthropometric data of lactating women and neonates across countries.

Variables	Total Sample (*n* = 128)	Montenegro (*n* = 55)	Serbia (*n* = 36)	North Macedonia (*n* = 37)
Lactating women				
Weight (kg)	76.2 ± 13.0	79.9 ± 11.7	70.7 ± 9.9	76.1 ± 15.7
Height (cm)	168.6 ± 6.3	170.3 ± 6.1	169.6 ± 5.2	165.2 ± 6.3
Body mass index (kg/m^2^)	26.8 ± 4.5	27.5 ± 3.4	24.6 ± 3.2	27.9 ± 6.1
Subjective assessment of dietary habits (%)				
Poor	1.6	1.9	2.8	78.4
Good	57.5	44.4	55.6	21.6
Very good	40.2	53.7	38.9	0.0
Do not know	0.8	0.0	2.8	0.0
DHA supplementation (%)				
No	68.8	49.1	66.7	100.0
Yes	31.3	50.9	33.3	0.0
Children at birth				
Weight (g)	3472.7 ± 440.6	3465.4 ± 410.7	3469.4 ± 339.7	3486.5 ± 564.5
Length (cm)	52.0 ± 1.3	53.2 ± 1.9	51.9 ± 1.8	50.6 ± 2.3

DHA—docosahexaenoic acid; descriptive data are presented as means and standard deviation or frequencies/percentages.

**Table 2:** Intake of *n*-3 PUFA and DHA from food sources.

Countries	Visit	Total *n*-3 PUFA (mg/Day)	*p* Values *	DHA (mg/Day)	*p* Values *
Total sample	V1 (*n* = 128)	91.3 (17.2–227.5)	0.383	3.0 (0.0–7.8)	0.099
	V2 (*n* = 115)	118.8 (17.2–245.1)		3.7 (0.0–11.1)	
Montenegro	V1 (*n* = 55)	48.9 (0.0–163.5)	0.517	2.1 (0.0–7.5)	0.657
	V2 (*n* = 42)	59.1 (0.0–158.8)		2.8 (0.0–7.5)	
Serbia	V1 (*n* = 36)	122.9 (26.1–290.5)	0.999	3.1 (0.0–8.4)	0.999
	V2 (*n* = 36)	122.9 (26.1–290.5)		3.1 (0.0–8.4)	
North Macedonia	V1 (*n* = 37)	135.3 (71.0–238.3)	0.100	4.8 (1.8–8.8)	<0.001
	V2 (*n* = 37)	186.9 (84.6–382.3)		9.7 (2.9–18.7)	

PUFA—polyunsaturated fatty acids; DHA—docosahexaenoic acid; descriptive data are presented as median (interquartile range). * Differences between visits were tested using the Wilcoxon test (*p* < 0.05).

**Table 3:** Correlation analysis of DHA intake and content in breast milk among unsupplemented and supplemented lactating women across countries.

Countries	Visit 1 (V1) (*n* = 128)				Visit 2 (V2) (*n* = 115)			
	Dietary Intake (mg/day)	Breast Milk (%)	r *	*p* Value	Dietary Intake (mg/day)	Breast Milk (%)	r *	*p* Value
Total sample (*n* = 128)	3.0 (0.0–7.8)	0.151 (0.111–0.210)	0.285	0.001 **	3.7 (0.0–11.1)	0.130 (0.094–0.190)	0.238	0.012 **
Unsupplemented women (V1, *n* = 88; V2, *n* = 84)	3.0 (0.0–7.3)	0.151 (0.112–0.202)	0.338	0.002 **	4.2 (0.0–11.5)	0.130 (0.100–0.190)	0.277	0.012 **
Supplemented women (V1, *n* = 40; V2, *n* = 31)	2.8 (0.3–8.2)	0.147 (0.111–0.262)	−0.255	0.308	3.7 (0.4–7.8)	0.120 (0.081–0.019)	0.588	0.044 **
Montenegro total (*n* = 55) (V1, *n* = 55; V2, *n* = 42)	2.1 (0.0–7.5)	0.138 (0.108–0.180)	0.226	0.096	2.8 (0.0–7.5)	0.101 (0.058–0.160)	0.150	0.344
Unsupplemented women (V1, *n* = 27; V2, *n* = 23)	1.0 (0.0–7.4)	0.119 (0.095–0.159)	0.445	0.020 **	2.7 (0.0–10.4)	0.100 (0.059–0.140)	0.414	0.050 **
Supplemented women (V1, *n* = 28; V2, *n* = 19)	2.8 (0.0–7.2)	0.142 (0.118–0.221)	−0.024	0.904	2.9 (0.2–6.4)	0.102 (0.057–0.175)	−0.265	0.273
Serbia total (*n* = 36) (V1, *n* = 36; V2, *n* = 36)	3.1 (0.0–8.4)	0.155 (0.080–0.205)	0.311	0.065	3.1 (0.0–8.4)	0.150 (0.120–0.240)	0.326	0.052
Unsupplemented women (V1, *n* = 24; V2, *n* = 24)	0.7 (0.0–5.1)	0.147 (0.085–0.180)	0.172	0.422	0.7 (0.0–5.1)	0.145 (0.120–0.245)	0.302	0.152
Supplemented women (V1, *n* = 12; V2, *n* = 12)	5.3 (1.0–12.0)	0.180 (0.041–0.320)	0.453	0.139	5.3 (1.0–12.0)	0.155 (0.120–0.210)	0.433	0.159
Northern Macedonia total (V1, *n* = 37; V2, *n* = 37) ^1^	4.8 (1.8–8.8)	0.181 (0.150–0.219)	0.207	0.240	9.7 (2.9–18.7)	0.147 (0.104–0.190)	0.230	0.190

DHA—docosahexaenoic acid; descriptive data are presented as median (interquartile range); ^1^ only unsupplemented lactating women; * Spearman correlation coefficient (** *p* < 0.05).

## Data Availability

Due to ethical and privacy restrictions, the datasets generated and analyzed during the current study are not publicly archived. Access to the de-identified data may be granted to researchers upon reasonable request and with the approval of the participating institutions.

## Supplementary Materials

The following supporting information can be downloaded at: https://www.mdpi.com/article/10.3390/nu18172761/s1, Supplementary Table S1. Complete fatty acid profile detected in human milk from lactating women in Serbia (SRB), Montenegro (MNE), and North Macedonia (MDK). A total of 40 fatty acids were identified by GC-FID across all three study sites: 17 saturated (SFA), 9 monounsaturated (MUFA), 3 trans (TFA), and 11 polyunsaturated (PUFA), of which 4 belong to the n-3 series. Results are expressed as wt% of total identified fatty acids (S = 100%). Fatty acids ordered by chain length and degree of unsaturation [39,40].

## Abbreviations

The following abbreviations are used in this manuscript:

ARA	Arachidonic Acid
BMI	Body Mass Index
DHA	Docosahexaenoic Acid
DRV	Dietary Reference Values
EFSA	European Food Safety Authority
EPA	Eicosapentaenoic Acid
PUFA	Polyunsaturated Fatty Acids
WHO	World Health Organization
