# Supplementary material for: Docosahexaenoic Acid (DHA) Deficiency in Breast Milk: A Cross-Sectional Study of Lactating Women in Serbia, Montenegro, and North Macedonia
Source: Nutrients. 2026 Aug 24;18(17):2761. doi: 10.3390/nu18172761 (PMC13567311; doi:10.3390/nu18172761)
Supplement: Supplementary file 1 [file nutrients-18-02761-s001.zip › nutrients-4317017-supplementary.pdf]

Supplementary Table S1. Complete fatty acid profile detected in human milk from lactating women in Serbia (SRB), Montenegro (MNE), and North Macedonia (MDK). A total of 40 fatty acids were identified by GC-FID across all three study sites: 17 saturated (SFA), 9 monounsaturated (MUFA), 3 trans (TFA), and 11 polyunsaturated (PUFA), of which 4 belong to the n-3 series. Results are expressed as wt% of total identified fatty acids (S = 100%). Fatty acids ordered by chain length and degree of unsaturation.

#	Systematic (IUPAC) Name	Common Name / Abbreviation	Shorthand (notation)	FA Class	SRB	MNE	MDK	Role / Biological Significance	n-3 PUFA sum	Total PUFA sum
SATURATED FATTY ACIDS (SFA)										
1	*Butanoic acid*	Butyric acid	C4:0	SFA	–	–	✓	Short-chain SFA; North Macedonia only	–	–
2	*Hexanoic acid*	Caproic acid	C6:0	SFA	✓	✓	✓	Short-chain SFA	–	–
3	*Octanoic acid*	Caprylic acid	C8:0	SFA	✓	✓	✓	Medium-chain SFA	–	–
4	*Decanoic acid*	Capric acid	C10:0	SFA	✓	✓	✓	Medium-chain SFA	–	–
5	*Undecanoic acid*	Undecylic acid	C11:0	SFA	–	✓	✓	Odd-chain SFA; trace levels	–	–
6	*Dodecanoic acid*	Lauric acid	C12:0	SFA	✓	✓	✓	Medium-chain SFA; antimicrobial properties	–	–
7	*Tridecanoic acid*	Tridecylic acid	C13:0	SFA	–	✓	✓	Odd-chain SFA; trace levels	–	–
8	*Tetradecanoic acid*	Myristic acid	C14:0	SFA	✓	✓	✓	Long-chain SFA	–	–
9	*Pentadecanoic acid*	Pentadecylic acid	C15:0	SFA	–	✓	✓	Odd-chain SFA; dairy biomarker	–	–
10	*Hexadecanoic acid*	Palmitic acid	C16:0	SFA	✓	✓	✓	Most abundant SFA in human milk; sn-2 position specific	–	–
11	*Heptadecanoic acid*	Margaric acid	C17:0	SFA	✓	✓	✓	Odd-chain SFA; ruminant-fat biomarker	–	–
12	*Octadecanoic acid*	Stearic acid	C18:0	SFA	✓	✓	✓	Long-chain SFA; precursor of oleic acid	–	–
13	*Eicosanoic acid*	Arachidic acid	C20:0	SFA	✓	✓	✓	Very-long-chain SFA	–	–
14	*Heneicosanoic acid*	Heneicosylic acid	C21:0	SFA	–	✓	–	Odd-chain SFA; trace; Montenegro only	–	–
15	*Docosanoic acid*	Behenic acid	C22:0	SFA	–	✓	✓	Very-long-chain SFA	–	–
16	*Tricosanoic acid*	Tricosylic acid	C23:0	SFA	–	✓	–	Odd-chain SFA; trace; Montenegro only	–	–
17	*Tetracosanoic acid*	Lignoceric acid	C24:0	SFA	–	✓	✓	Very-long-chain SFA	–	–
MONOUNSATURATED FATTY ACIDS (MUFA)										
18	*cis-9-Tetradecenoic acid*	Myristoleic acid	C14:1n-5	MUFA	–	✓	✓	Reported in Montenegro and North Macedonia	–	–
19	*cis-10-Pentadecenoic acid*	Pentadecenoic acid	C15:1n-5	MUFA	–	✓	–	Trace; Montenegro only	–	–
20	*cis-9-Hexadecenoic acid*	Palmitoleic acid	C16:1n-7	MUFA	✓	✓	✓	Lipogenic index marker; de novo synthesis	–	–
21	*cis-10-Heptadecenoic acid*	Heptadecenoic acid	C17:1n-7	MUFA	–	✓	✓	Odd-chain MUFA	–	–
22	*cis-9-Octadecenoic acid*	Oleic acid	C18:1n-9	MUFA	✓	✓	✓	Most abundant FA in human milk; reflects dietary fat	–	–
23	*cis-11-Octadecenoic acid*	Vaccenic acid (cis)	C18:1n-7 (cis-11)	MUFA	–	✓	✓	Ruminant-derived; minor MUFA	–	–
24	*cis-11-Eicosenoic acid*	Gondoic acid	C20:1n-9 (cis-11)	MUFA	–	✓	✓	–	–	–
25	*cis-13-Docosenoic acid*	Erucic acid	C22:1n-9 (cis-13)	MUFA	–	✓	✓	Trace; rapeseed oil dietary marker	–	–
26	*cis-15-Tetracosenoic acid*	Nervonic acid	C24:1n-9 (cis-15)	MUFA	–	✓	✓	Sphingomyelin component; myelin synthesis	–	–
TRANS FATTY ACIDS (TFA)										
27	*trans-9-Octadecenoic acid*	Elaidic acid	C18:1n-9 (trans-9)	TFA	✓	✓	✓	Industrial trans-fatty acid; dietary marker	–	–
28	*trans-11-Octadecenoic acid*	Vaccenic acid (trans)	C18:1n-7 (trans-11)	TFA	–	✓	–	Ruminant-derived TFA; Montenegro only	–	–
29	*trans-9,12-Octadecadienoic acid*	Trans-linoleic acid	C18:2n-6 (trans-9,12)	TFA	–	✓	✓	Non-conjugated TFA	–	–
POLYUNSATURATED FATTY ACIDS (PUFA)										
30	*cis-9,12-Octadecadienoic acid*	Linoleic acid (LA)	C18:2n-6 (cis-9,12)	PUFA	✓	✓	✓	Essential FA; most abundant PUFA in human milk	–	✓
31	*all-cis-6,9,12-Octadecatrienoic acid*	Gamma-linolenic acid (GLA)	C18:3n-6 (cis-6,9,12)	PUFA	–	✓	✓	Elongation/desaturation product of LA	–	✓
32	*cis-11,14-Eicosadienoic acid*	Eicosadienoic acid	C20:2n-6 (cis-11,14)	PUFA	–	✓	✓	–	–	✓
33	*all-cis-5,8,11-Eicosatrienoic acid*	Mead acid †	C20:3n-9 (cis-5,8,11)	PUFA	–	✓	–	Marker of essential FA deficiency (n-9 series); Montenegro only	–	✓
34	*all-cis-8,11,14-Eicosatrienoic acid*	DGLA (Dihomo-γ-linolenic)	C20:3n-6 (cis-8,11,14)	PUFA	✓	–	✓	Series-1 prostaglandin precursor; anti-inflammatory	–	✓
35	*all-cis-5,8,11,14-Eicosatetraenoic acid*	Arachidonic acid (ARA)	C20:4n-6 (cis-5,8,11,14)	PUFA	–	✓	✓	Critical for infant brain/retina; relatively diet-independent	–	✓
36	*cis-13,16-Docosadienoic acid*	Docosadienoic acid	C22:2n-6 (cis-13,16)	PUFA	–	✓	✓	–	–	✓
► n-3 Polyunsaturated Fatty Acids (ω-3) — included in total n-3 PUFA sum										
37	*all-cis-9,12,15-Octadecatrienoic acid*	Alpha-linolenic acid (ALA)	C18:3n-3 (cis-9,12,15)	PUFA	✓	✓	✓	Essential FA; parent of metabolic n-3 series	✓	✓
38	*all-cis-11,14,17-Eicosatrienoic acid*	Eicosatrienoic acid (ETE)	C20:3n-3 (cis-11,14,17)	PUFA	✓	✓	–	Intermediate in ALA→EPA→DHA pathway	✓	✓
39	*all-cis-5,8,11,14,17-Eicosapentaenoic acid*	Eicosapentaenoic acid (EPA)	C20:5n-3 (cis-5,8,11,14,17)	PUFA	–	✓	✓	Long-chain n-3 PUFA; anti-inflammatory eicosanoids	✓	✓
**40**	**all-cis-4,7,10,13,16,19-Docosahexaenoic acid**	**Docosahexaenoic acid (DHA) ★**	**C22:6n-3 (cis-4,7,10,13,16,19)**	PUFA	✓	✓	✓	**PRIMARY OUTCOME — threshold ≥0.30 wt% for optimal infant neurodevelopment**	✓	✓

n-3 PUFA sum = ALA + ETE + EPA + DHA. DHA threshold: ‡0.30 wt% required for optimal infant neurocognitive and visual development [39,40]. FAMEs prepared by alkaline transesterification (ISO 12966-1, -2, -4); separated on Rtx-2330 capillary column (105 m × 0.25 mm × 0.2 µm; Shimadzu Nexis GC-2030). Identification:primary standard at all three laboratories — Supelco 37 Component FAME Mix, Trace CERT® (CRM47885, Sigma-Aldrich/Supelco), C4:0–C22:6n-3. Supplementary standards at North Macedonia: C4–C24 Even Carbon Saturated FAMEs (49453-U), Linoleic Acid Methyl Ester Mix cis/trans (CRM47791), Linolenic Acid Methyl Ester Mix cis/trans (CRM47792) (all Supelco). Montenegro: Restek Food Industry FAME Mix 37 components (Cat. No. 35077) + Restek Trans Fat Reference Standard (Cat. No. 35629). Serbia: FAME standards per SRPS EN ISO 12966 (C4–C24 range); undetected fatty acids were below the limit of quantification. Results: x i = (A i / SA) × 100. Inter-laboratory differences in the number of fatty acids quantified above the LOQ may introduce a small systematic difference in the wt% denominator; cross-country comparisons should be interpreted accordingly. 4 = detected (‡1participant); – = not reported/below LOQ. SRB = Serbia; MNE = Montenegro; MDK = North Macedonia. Row shading: grey = SFA; blue = MUFA; orange = TFA; green = PUFA n-6; light gold = n-3 PUFA; bright gold = DHA (H primary outcome).
